# A Fortune for Barbers

**Published:** 1875-06

**Authors:** 


### A FORTUNE FOR BARBERS.

We are anxious to make a fortune for one or more of the barbers in all the cities and large towns in the United States. We see plainly that we have the power to do this, provided we find the right sort of barbers to act as recipients of the large income which we can influence, and we question if we have any right to withhold it; so we determine to make our intentions public, and to seek out the parties who are to be thus largely benefitted at our hands.

To secure the wealth which we have power to bestow, very little is needed on the part of the barbers. Nothing, in fact, is required which would be looked upon as onerous by any decent, well-bred man or woman. The simple qualifications which we insist upon, are only such as belong to refined humanity, the world over; such as are generally lacking among savages and persons in the depths of degradation. It would seem, therefore, that qualifications of this moderate type should be easily found in a thousand directions.

All we ask, before turning on the golden shower, is proof that those who handle the heads and fumble the faces of mankind, and wish to compete for the fortune alluded to, practise reasonable neatness in the discharge of their daily duties. We shall merely wish to be satisfied that the barber who applies for the boundless wealth indicated, has, for twelve months past, never manipulated a human cranium without first carefully washing from his hands the excreta and other proofs of contact, accumulated from the last customer; that he has not blown a foul breath upon the face of any sufferer; that he has cleansed his combs and brushes by daily immersion in an alkaline fluid strong enough to convert human oils into soap and render them non-offensive; that he has avoided the use of tobacco and other unclean things in order to keep his person sweet; that he has taken a daily bath in order to save his patrons from nausea and disgust; that he has allowed no alcoholic compounds to impart disagreeable odors to his exhalations, and make him an unclean and sickening creature to associate with;—in short, that he has led a decent and cleanly life. These are the simple conditions which we insist upon, and no sensible person will dare assert that anything less than this should be demanded of those who manipulate the lips and cheeks and brows of human beings.

Will there be much of a rush for the fortune which is in store for men of decency engaged in shaving and hair-dressing? Oh, no. Nobody will apply for the fortune, because nobody can present the required conditions. Barbers are dirty creatures, one and all. Not one in a hundred of them brush their teeth after each meal to secure a sweet and wholesome breath. Not one in a thousand washes the dirt of one person from his hands before touching the face of another; not one in a million keeps himself and his tools in a condition to make contact therewith anything else than a thing to be abhorred.

Nobody comes as near to us as our barbers; no class has equal power to make us sick and sad and savage; and no class avails itself more completely of all its opportunities. It will be hard to change the natures of these unclean savages, but it can be done. Its accomplishment rests only with the people who are compelled to patronize the shops.

Let our readers take this magazine to the nearest barber, and say that hereafter absolute neatness must be practised upon them, or their custom shall be withheld. Let them boldly assert that they will not allow a breath laden with the pestilence of whisky and tobacco and decomposing food, and foul teeth, and horribly diseased secretions, to be blown in their faces. Let them declare that the odors and emanations of perhaps a vicious and diseased predecessor in the barber's chair shall be removed from the hands and person of the operator, with scrupulous care, abundant soap, and copious supplies of pure water. Let them rebel at soiled napkins and contaminated brushes and combs. And let us hope that the barber who is not altogether barbarous, will change his indecent course and make himself worthy of near association and contact with human beings. From being the dirtiest of humanity, let him be the cleanest, as he should be by virtue of the close proximity which is permitted him with some refined and sensitive people. To the barbers who are foremost in this much needed reform, fortune is sure as fate; and we promise the entire weight of our influence to those who will strive for a higher life in the direction of that cleanliness, which is such an enormous stride toward Godliness.

---

Little girls, to grow up and be healthy and rosy, must have plenty of out-door exercise.

